# Polymerase Chain Reaction on Respiratory Tract Specimens of Immunocompromised Patients to Diagnose *Pneumocystis* Pneumonia: A Systematic Review and Meta-analysis

**DOI:** 10.1093/cid/ciae239

**Published:** 2024-06-11

**Authors:** Lottie Brown, Riina Rautemaa-Richardson, Carlo Mengoli, Alexandre Alanio, Rosemary A Barnes, Stéphane Bretagne, Sharon C A Chen, Catherine Cordonnier, J Peter Donnelly, Werner J Heinz, Brian Jones, Lena Klingspor, Juergen Loeffler, Thomas R Rogers, Eleanor Rowbotham, P Lewis White, Mario Cruciani

**Affiliations:** Institute of Infection and Immunity, St George's University and St Georges University Hospitals NHS Foundation Trust, London, United Kingdom; Mycology Reference Centre Manchester and Department of Infectious Diseases, Manchester Academic Health Science Centre, Wythenshawe Hospital, Manchester University NHS Foundation Trust and Division of Evolution, Infection and Genomics, Faculty of Biology, Medicine and Health, University of Manchester, United Kingdom; Department of Infectious, Parasitic and Immune-Mediated Diseases, Instituto Superiore Di Sanita, Rome, Italy; Unité de Mycologie Moléculaire, Institut Pasteur, Paris, France; Department of Infection, Immunity and Biochemistry and School of Medicine, University of Cardiff, United Kingdom; Université Paris Cité, Parasitology-Mycology Laboratory, Hôpital Saint-Louis, APHP, Paris, France; Centre for Infectious Diseases and Microbiology Laboratory Services, Institute of Clinical Pathology and Medical Research, New South Wales Health Pathology, Westmead Hospital, Westmead, Australia; Haematology and Stem Cell Transplant Department, Henri Mondor Hospital, and University Paris-Est-Créteil, Créteil, France; Fungal PCR Initiative, a working group of the International Society of Human and Animal Mycology, Verona, Italy; Med. Clinic II, Caritas Hospital Bad Mergentheim, Germany; Institute of Infection, Immunity and Inflammation, University of Glasgow, United Kingdom; Karolinska Institutet, Department of Laboratory Medicine, Karolinska University Hospital Huddinge, Stockholm, Sweden; Medizinische Klinik II, Labor WÜ4i, Universitätsklinikum Würzburg, Germany; Discipline of Clinical Microbiology, Trinity College Dublin, St James’s Hospital Campus, Dublin, Ireland; Mycology Reference Centre Manchester and Department of Infectious Diseases, Manchester University, Manchester University NHS Foundation Trust, Wythenshawe Hospital, Manchester; Public Health Wales Mycology Reference Laboratory, Public Health Wales Microbiology Cardiff, University Hospital of Wales, and Centre for Trials Research/Division of Infection and Immunity, Cardiff University, United Kingdom; Fungal PCR Initiative, a working group of the International Society of Human and Animal Mycology, Verona, Italy

**Keywords:** polymerase chain reaction, pneumocystis, PCP, meta-analysis, systematic review

## Abstract

**Background:**

This meta-analysis examines the comparative diagnostic performance of polymerase chain reaction (PCR) for the diagnosis of *Pneumocystis* pneumonia (PCP) on different respiratory tract samples, in both human immunodeficiency virus (HIV) and non-HIV populations.

**Methods:**

A total of 55 articles met inclusion criteria, including 11 434 PCR assays on respiratory specimens from 7835 patients at risk of PCP. QUADAS-2 tool indicated low risk of bias across all studies. Using a bivariate and random-effects meta-regression analysis, the diagnostic performance of PCR against the European Organisation for Research and Treatment of Cancer–Mycoses Study Group definition of proven PCP was examined.

**Results:**

Quantitative PCR (qPCR) on bronchoalveolar lavage fluid provided the highest pooled sensitivity of 98.7% (95% confidence interval [CI], 96.8%–99.5%), adequate specificity of 89.3% (95% CI, 84.4%–92.7%), negative likelihood ratio (LR^−^) of 0.014, and positive likelihood ratio (LR^+^) of 9.19. qPCR on induced sputum provided similarly high sensitivity of 99.0% (95% CI, 94.4%–99.3%) but a reduced specificity of 81.5% (95% CI, 72.1%–88.3%), LR^−^ of 0.024, and LR^+^ of 5.30. qPCR on upper respiratory tract samples provided lower sensitivity of 89.2% (95% CI, 71.0%–96.5%), high specificity of 90.5% (95% CI, 80.9%–95.5%), LR^−^ of 0.120, and LR^+^ of 9.34. There was no significant difference in sensitivity and specificity of PCR according to HIV status of patients.

**Conclusions:**

On deeper respiratory tract specimens, PCR negativity can be used to confidently exclude PCP, but PCR positivity will likely require clinical interpretation to distinguish between colonization and active infection, partially dependent on the strength of the PCR signal (indicative of fungal burden), the specimen type, and patient population tested.

## Teaser

This meta-analysis demonstrated very high diagnostic performance of PCR assays when used as screening tests for *Pneumocystis* pneumonia in high-risk patient groups, regardless of HIV status. qPCR on bronchoalveolar lavage fluid samples provided optimal sensitivity, closely followed by qPCR on induced sputum, while also providing adequate specificity.

## Graphical abstract

**Graphical abstract:**
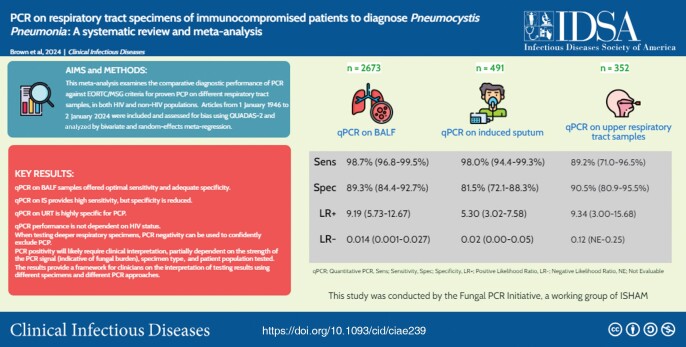
This graphical abstract is also available at Tidbit: https://tidbitapp.io/tidbits/pcr-on-respiratory-tract-specimens-of-immunocompromised-patients-to-diagnose-pneumocystosis-pcp-a-systematic-review-and-meta-analysis-89aa7a50-efbc-4ebb-8f20-c17d207e8d8e?utm_campaign=tidbitlinkshare&utm_source=ITP

*Pneumocystis* pneumonia (PCP) is typically seen in patients with advanced human immunodeficiency virus (HIV) infection, but rates are increasing in other groups of immunocompromised patients, including patients with solid tumors, hematological malignancies, primary immunodeficiencies or autoimmune and inflammatory conditions requiring immunomodulating therapies, and stem cell and solid organ transplant recipients [1–3]. In contrast to PCP in HIV/AIDS, the disease course in these immunocompromised groups can be characterized by an abrupt onset of respiratory failure, rapid deterioration, and higher mortality rate, despite lower fungal burdens [4]. There is also a growing number of reports of PCP in patients with contaminant viral infections, including influenza and coronavirus disease 2019 [5, 6].

The 2020 revision of the European Organisation for Research and Treatment of Cancer–Mycoses Study Group (EORTC/MSG) definitions of invasive fungal diseases categorizes the diagnosis of PCP as either proven or probable disease dependent on the level of mycological evidence [7]. As with all other invasive mycoses, proven PCP diagnosis has required a combination of clinical and radiological features plus detection of *Pneumocystis jirovecii* by microscopy of respiratory tract specimens [8]. Sensitivity of microscopy is dependent on operator skills and fungal burden, and false negatives may occur in patients with low fungal burden, such as those who are HIV negative [4]. Currently, polymerase chain reaction (PCR) only contributes to a diagnosis of probable PCP, primarily due to limited standardization and absence of clear criteria for the interpretation of results to distinguish *Pneumocystis* colonization from infection. A wide range of quantitative PCR (qPCR) assays have been developed with the capacity to quantify fungal burdens, and their high sensitivity means even very low fungal loads may be detected in either bronchoalveolar lavage fluid (BALF) or induced sputum (IS) samples. Quantification of fungal load is key when interpreting qPCR results and differentiating between colonization and active infection [9]. Assay-specific thresholds have attempted to distinguish between colonization and infection but have not been adjusted according to disease or specimen type/quality and require standardization, making comparisons between centers/assays difficult [10]. Although several meta-analyses have investigated the performance of PCR of various sampling approaches for diagnosis of PCP, they are limited by the inclusion of lower-quality studies (case-controls), absence of comparison with an appropriate reference standard, exclusion of non-English-language studies, and failure to include different specimen types, PCR methods, and HIV and non-HIV populations (Supplementary Material 1) [11–14]. The aim of this study was to examine the comparative diagnostic performance of PCR and sampling approaches, in both HIV and non-HIV populations with proven PCP, with a view to inform clinicians on the best strategy for PCP diagnosis and accurate interpretation of PCR results.

## METHODS

This study was conducted by the *Pneumocystis* PCR clinical working party of the Fungal PCR Initiative, a working group of the International Society for Human and Animal Mycology.

For this systematic review and meta-analysis, a standardized search was conducted from 1 January 1946 to 2 January 2024 using PubMed, Scopus, Embase, Cumulative Index to Nursing and Allied Health Literature, and Cochrane (search terms in Supplementary Material 2), without language restrictions. Additional relevant articles from the reference sections were also reviewed.

Original studies that used PCR-based methods on any respiratory specimens of humans were analyzed for eligibility. Studies were eligible if (1) PCR results were compared with the reference diagnosis made using standard laboratory methods in line with the EORTC/MSGERC definition of proven PCP; (2) sufficient information was provided to assess the robustness of the diagnosis; (3) results were reported as false positive, true positive, false negative, and true negative, or these data could be derived from the study if not specifically stated; and (4) evaluation of the test(s) was performed in groups of individuals at high risk for PCP where there is clinical suspicion of the disease. Studies retrospectively evaluating specimens from a group of patients known to have PCP, and from a separate group of subjects without evidence of disease, were regarded as case-control studies and excluded.

Pairs of authors independently screened articles for eligibility, selected articles for full-text review, extracted data from the included studies, evaluated the methodological quality of eligible papers according to the Standards for Reporting of Diagnostic Accuracy (STARD), and assessed risk of bias by use of the Quality Assessment of Diagnostic Accuracy Studies-2 (QUADAS-2) tool, with any disagreements resolved by a third author blinded to previous reviews [15, 16]. The International Prospective Register of Systematic Reviews (PROSPERO) protocol is available online (https://www.crd.york.ac.uk/prospero/display_record.php?ID=CRD42018087812).

For included studies, either individual data or summary estimates of sample size and the number of true-positive, true-negative, false-positive, and false-negative results of PCR in each study were extracted. The sensitivity, specificity, positive likelihood ratio (LR^+^), and negative likelihood ratio (LR^−^) of PCR tests and the 95% confidence intervals (CIs) were calculated. Positive predictive values (PPVs) and negative predictive values (NPVs) were also calculated. The main statistical approach consisted of a logistic mixed-model procedure designed to estimate sensitivity and specificity as distinct results (a bivariate model) [17]. Predictors were included to evaluate their effect on PCR assay performance and configure a meta-regression model. Predictors included underlying disease (HIV vs non-HIV), type of sample (BALF, IS, or upper respiratory tract [URT]) and PCR method (conventional PCR [cPCR] or qPCR). HIV prevalence was preliminarily judged to be weakly or not influential, so the model was simplified by omitting this potential covariate. The software employed was Stata 17.0 “melogit.” Diagnostics odds ratio (DOR), a measure of overall diagnostic accuracy incorporating both sensitivity and specificity, was also calculated from the coefficients of the bivariate model with the method of linear combination (Stata command “lincom”) [17]. Heterogeneity was evaluated by the main model, which consisted of a random-effects meta-regression analysis. A second approach, separate from the former, using methods not supporting meta-regression, were applied to subgroups created in order to achieve additional insight on the role of some of the context variables. For this approach, Stata “metandi” was used to obtain bivariate plot figures for sample = BALF, sample = IS, sample = URT, when PCR was qPCR or cPCR. Heterogeneity was evaluated by visual inspection of forest plots of sensitivity and specificity of PCR by sample type. We did not evaluate publication bias because there is no appropriate test with adequate statistical power to reliably assess publication bias in the context of diagnostic test accuracy systematic reviews [18].

## RESULTS

**Figure 1.**
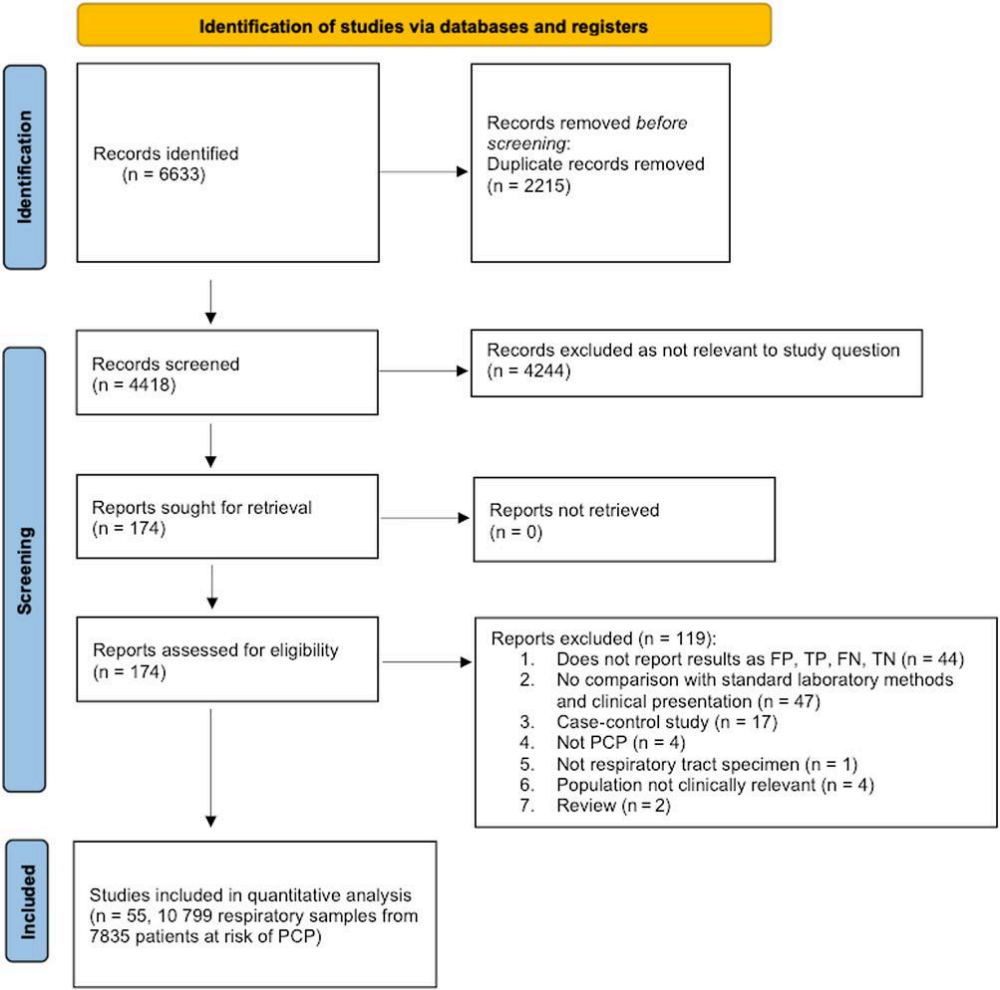
Study selection. Abbreviations: FN, false negative; FP, false positive; PCP, *Pneumocystis* pneumonia; TN, true negative; TP, true positive.

Of the 4418 references identified, 174 potentially relevant articles were selected for full-text review. After full-text review, 119 studies were excluded for various reasons (Figure 1). Therefore, 55 studies published between 1991 and 2023 met the inclusion criteria and were included in the systematic review and meta-analysis (Supplementary Material 3).

A total of 11 434 PCR assays using 10 799 non-duplicate respiratory specimens from 7835 patients at risk of PCP were included in the meta-analysis. The studies were from 21 different countries and most were single center in design (n = 48 [84%]). The type of microscopy used in the reference standard varied from cytochemical staining (Giemsa ± Grocott methenamine silver ± toluidine blue) (n = 19) to direct detection by immunofluorescence assay (IFA) (n = 25) or both (n = 11). Study characteristics are summarized in Supplementary Material 4 and 5.

Around a third (n = 2723 [34.8%]) of patients were reported to be HIV positive, 36.0% (n = 2822) of patients were HIV negative, and HIV status was not specified in 29.2% (n = 2290). The most common cause of immunocompromise after HIV infection was hematological malignancy (n = 1000 [12.8%]), solid organ transplant (n = 463 [5.9%]), immunosuppressive medication (corticosteroids, cytostatics, biologic agents, monoclonal antibodies, calcineurin and noncalcineurin inhibitors) (n = 428 [5.5%]), and solid tumors (n = 351 [4.8%]).

The prevalence of proven PCP across all studies ranged from 1.1% to 81.8%, with a mean prevalence of 20.7%. Specimens tested by PCR were BALF (n = 7199 [66.7%]), followed by IS (n = 2309 [21.4%]), oral washes (n = 488 [4.5%]), nasopharyngeal aspirates (n = 301 [2.8%]), and other (n = 502 [4.6%]). Sufficient data to allow for subgroup analysis of diagnostic performance of PCR according to sample type were not available in a third of respiratory specimens (n = 4104 [38.0%]). There were variations in the PCR formats used: qPCR was used in 27 studies, qualitative gel-based cPCR was used in 26 studies, and both cPCR and qPCR were used in 2 studies. Only 12 articles specified quantification cycle values for optimal diagnosis of PCP based on receiver operating characteristic analysis. The threshold for diagnosing PCP varied from 22 to 38 cycles across studies. Details of the PCR techniques used are summarized in Supplementary Material 4.

A total of eighty-six 2 × 2 tables reporting true-positive, false positive, false-negative, and true-negative cases were obtained from the 55 primary trials. QUADAS-2 assessment demonstrated that all included studies were of good or acceptable quality and low risk of bias. Concerns regarding applicability were rarely found (Supplementary Material 6 and 7).

**Figure 2.**
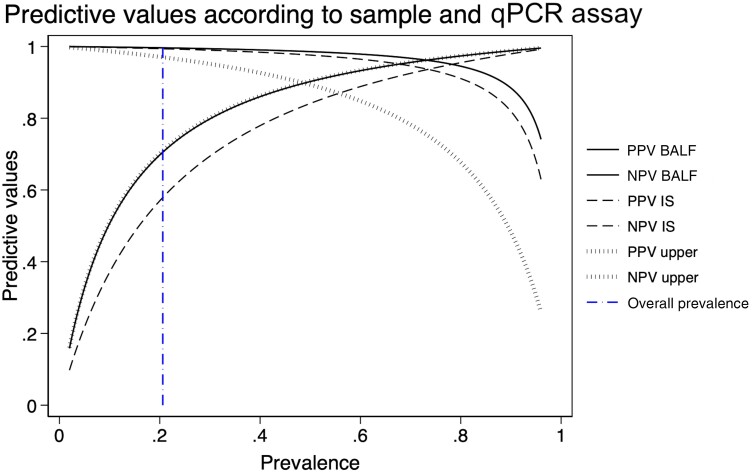
Positive predictive values (PPVs) and negative predictive values (NPVs) presented as probability curves for quantitative polymerase chain reaction on each sample type. PPVs increase and NPVs decrease with prevalence. The vertical dash-dotted line indicates the overall prevalence of *Pneumocystis* pneumonia in the data set (20.7%). Abbreviations: BALF, bronchoalveolar lavage fluid; IS, induced sputum; NPV, negative predictive value; PPV, positive predictive value; qPCR, quantitative polymerase chain reaction.

Sensitivity and specificity of PCR according to specimen type and PCR technique used is summarized in Table 1. There was no difference in the proportions of HIV and non-HIV patients among the cohorts of patients tested by cPCR or qPCR. The sensitivity, specificity, and related DOR values in the overall analysis were high. qPCR on BALF provided the highest pooled sensitivity of 98.7% (95% CI, 96.8%–99.5%), adequate specificity of 89.3% (95% CI, 84.4%–92.7%), a high DOR of 635, LR^−^ of 0.014, and LR^+^ of 9.19. qPCR on IS provided similarly high sensitivity of 98.0% (95% CI, 94.4%–99.3%), reduced specificity of 81.5% (95% CI, 72.1%–88.3%), a DOR of 217, LR^−^ of 0.024, and reduced LR^+^ of 5.30. qPCR on URT samples provided a reduced sensitivity of 89.2% (95% CI, 71.0%–96.5%), high specificity of 90.5% (95% CI, 80.9%–95.5%), the lowest DOR of 78, LR^−^ of 0.120, and LR^+^ of 9.34. Across all specimen types, the sensitivity of qPCR was greater than that of cPCR, while the specificity of qPCR was lower than that of conventional PCR. There was no significant difference in sensitivity and specificity of PCR according to HIV status of patients enrolled in the trial (z = 0.39, *P* = .698) (Supplementary Material 8 and 9). PPVs and NPVs were also calculated to understand how diagnostic accuracy may change according to disease prevalence (Figure 2).

**Table 1.** Sensitivity, Specificity, and Diagnostic Odds Ratio of Polymerase Chain Reaction (PCR) Test According to PCR Technique

Sample	Sensitivity (95% CI)	Specificity (95% CI)	DOR (95% CI)	LR^+^ (95% CI)	LR^−^ (95% CI)
BALF samples					
qPCR (n = 2673)	0.987 (.968–.995)	0.893 (.844–.927)	635 (269–1498)	9.194 (5.727–12.661)	0.014 (.001–.027)
cPCR (n = 2254)	0.972 (.932–.988)	0.954 (.930–.970)	710 (305–1652)	21.178 (12.438–29.918)	0.30 (.004–.056)
IS samples					
qPCR (n = 491)	0.980 (.944–.993)	0.815 (.721–.883)	217 (78–601)	5.303 (3.024–7.583)	0.024 (.000–.049)
cPCR (n = 590)	0.956 (.887–.984)	0.917 (.866–.950)	243 (90–656)	11.511 (5.985–17.036)	0.047 (.001–.094)
URT samples					
qPCR (n = 352)	0.892 (.710–.965)	0.905 (.809–.955)	78 (26–238)	9.340 (2.997–15.682)	0.120 (NE–.245)
cPCR (n = 512)	0.787 (.502–.931)	0.960 (.911–.982)	87 (27–284)	19.424 (5.358–33.490)	0.222 (NE–.446)

Sensitivity, specificity, DORs, and positive and negative likelihood ratios with 95% CI calculated from the coefficients of the binomial regression model. In regard to human immunodeficiency virus (HIV) versus non-HIV status, there was no significant difference in the cohorts of patients tested by cPCR or qPCR or by specimen types tested. Numbers of samples (n) where analysis of sensitivity/specificity was performed by sample type are shown. “NE” (not evaluable) appears when the corresponding results obtained by the calculating algorithm had a negative sign, which is impossible as the LRs are ratios between 2 positive numbers (proportions). This fact is due to the use of an approximate method to calculate the 95% CIs of the LRs, the “delta method.”

Abbreviations: BALF, bronchoalveolar lavage fluid; CI, confidence interval; cPCR, conventional polymerase chain reaction; DOR, diagnostic odds ratio; IS, induced sputum; LR^+^, positive likelihood ratio; LR^–^, negative likelihood ratio; NE, not evaluable; qPCR, quantitative polymerase chain reaction; URT, upper respiratory tract.

In the logistic regression model, IS specimens had higher rate of positive results compared to BALF samples (z = 2.87, *P* = .004), although the higher frequency of PCR positivity on IS was a result of its lower specificity compared to BALF (z = −2.52, *P* = .012), leading to a higher probability of generating false positives. While the main analysis was based on a multivariate mixed-model logistic regression, a bivariate meta-analysis of diagnostic accuracy on BALF, IS, and URT specimens was also performed (Supplementary Material 10).

**Figure 3.**
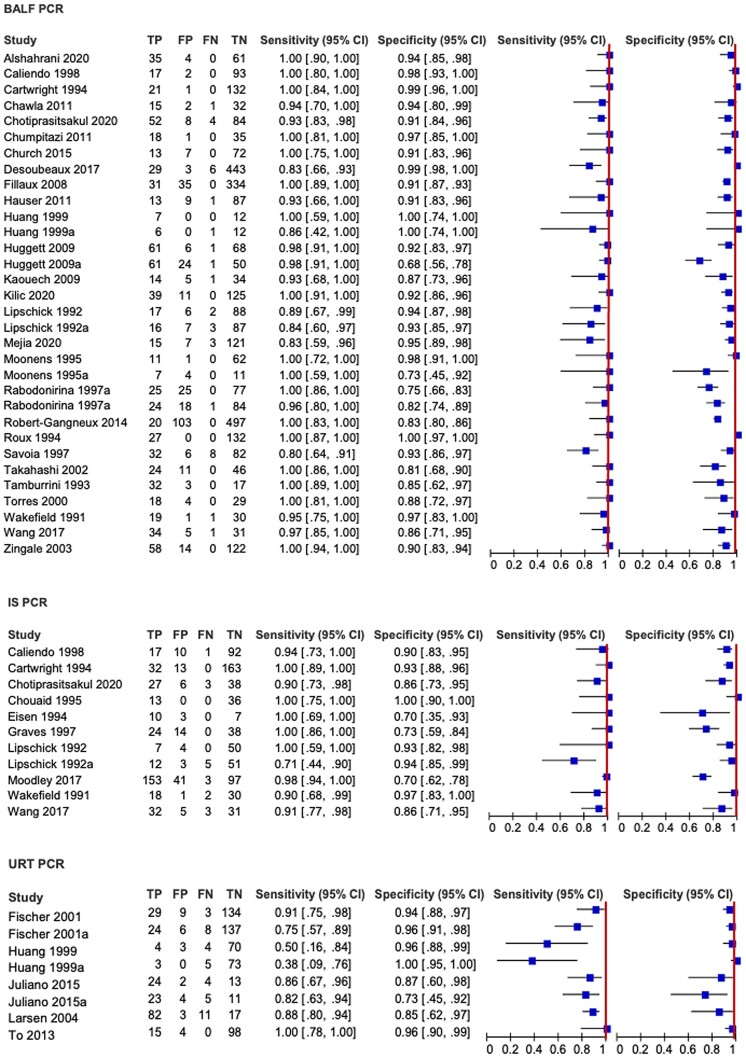
Forest plots for sensitivity and specificity of polymerase chain reaction by sample type. Heterogeneity was assessed by visual inspection of forest plots of sensitivity and specificity and through visual examination of the receiver operating characteristic plot of the raw data. Heterogeneity was further investigated by exploring the effects of several study-level covariates by random-effects meta-regression analysis. Abbreviations: BALF, bronchoalveolar lavage fluid; CI, confidence interval; FN, false negative; FP, false positive; IS, induced sputum; PCR, polymerase chain reaction; TN, true negative; TP, true positive; URT, upper respiratory tract.

Forest plots show moderate variability of the effect sizes and related CIs of individual studies, with specificity being more heterogeneous than sensitivity (Figure 3). The covariates best explaining heterogeneity of the overall analysis were the type of specimens evaluated and PCR methods, while HIV infection status had no relevant impact on the sensitivity nor specificity.

## DISCUSSION

There have been 4 meta-analyses [11–14] published previously with comparable pooled sensitivity and specificity of PCR on respiratory tract specimens (Supplementary Material 1). This meta-analysis presents a rigorous and comprehensive analysis of the comparative diagnostic performance of both cPCR and qPCR and various sampling approaches, in both HIV and non-HIV populations, including over 10 000 samples from exclusively high-quality, cohort studies.

These data confirm that qPCR on BALF and IS provides optimal sensitivity and adequate specificity to be recommended tests for the diagnosis of PCP, with IS providing a less invasive, less technically demanding, and lower-risk approach to sampling, which is important given that patients with PCP are often critically unwell and incidence is greatest in resource-limited settings. Although the sensitivity of qPCR outperformed cPCR across all samples, the sensitivity of cPCR is still high when testing lower respiratory tract samples. While this approach may be acceptable where qPCR is not readily available (eg, in resource-limited settings), the technical benefits associated with qPCR (quantification of fungal burden, reduced opportunity for contamination, identification of mutations potentially associated with resistance to PCP therapy) preclude the use of cPCR when qPCR is an option. While the specificity of qPCR was lower than that of conventional PCR across all sample types, the lower specificity and higher rates of qPCR “false positives” is likely due to its very high sensitivity, which allows for detection of very low fungal burdens that reflect colonization/contamination rather than genuine infection. While this was not overly represented by increased clinical sensitivity when testing lower respiratory tract samples, this could reflect the fact that the higher burdens associated with PCP are adequately detected by cPCR methods, despite their potentially inferior analytical sensitivity. When testing URT samples, where the available fungal burden is generally lower, the sensitivity of qPCR was superior to that of cPCR, while specificity was comparable (Table 1).

In the logistic regression model of all specimens, PCR on IS samples had higher sensitivity compared to BALF samples, while the coefficient analysis of sample and disease indicated a lower specificity, or a higher probability of finding false positives, perhaps attributable to detecting colonization rather than infection. Through use of a bronchoscope, BALF sampling is focused to a specific area, whereas the IS procedure potentially provides a broader sampling range and so may be more likely to detect colonization of the bronchial tree. Similarly, the anatomical sample range of URT samples is focused to a specific area and subsequently less prone to detecting false positives. While PCR positivity of URT specimens was historically thought to reflect patient colonization, it is now thought to reflect higher burdens in the lower respiratory tract and diagnostic accuracy is borne out by the higher specificity for PCR when testing URT specimens, particularly in HIV patients where fungal burdens are typically high [19]. Among other patient groups, such as those with underlying lung disease like chronic obstructive pulmonary disease, where prevalence of *Pneumocystis* colonization is high (ranging between 10% and 55% in a recent systematic review), a positive PCR result on URT specimen should be interpreted with caution [20].

Forest plots revealed moderate variability of the effect sizes and related CIs of individual studies, with specificity being more heterogeneous than sensitivity, most apparent in URT samples, possibly due to variations in sampling approach (nasopharyngeal aspirates, oral washes) or differences in underlying cause of immunocompromise in the populations sampled, which was not reported in 3 of the 10 studies presenting PCR on URT. Sensitivity of PCR on URT specimens is expected to be higher in populations with HIV, where fungal burdens in the lower respiratory tract are typically very high. Although our meta-analysis found no significant difference in sensitivity and specificity of PCR according to HIV status across all sample types, a recent meta-analysis by Senécal et al concluded that performance of PCR of IS was superior in HIV groups [14]. The discrepancy in these findings is possibly due to differences in inclusion criteria studies and pooling of qPCR and cPCR assays in the meta-analysis by Senécal et al. Data for non-HIV groups were limited, particularly for URT samples.

These data do not consider the burden of fungal disease as determined by an individual positive qPCR test, where higher burdens are generally associated with an increased likelihood of disease compared to colonization. The findings indicate that qPCR tests show very high diagnostic performance when used as screening tests for PCP in high-risk patient groups, where negativity of deeper respiratory tract specimens can be used to confidently exclude PCP. PCR positivity will likely require clinical interpretation, partially dependent on the strength of the PCR signal (indicative of fungal burden), the specimen type, and patient population tested. However, this multicomponent approach is commonplace in medical mycology, where near perfect diagnostic tests are rare with few exceptions and combining multiple laboratory diagnostic tests with clinical evidence typical of disease is common practice. Detection of serum (1–3)-β-D-glucan (BDG) may be combined with PCR to improve diagnostic accuracy, and PCP PCR positivity combined with a BDG >200 pg/mL generated 100% specificity for the diagnosis of PCP [21].

This review has several limitations. First, only a small number of studies provided data on URT samples, which may have limited the power to assess their diagnostic performance and necessitated pooling of nasopharyngeal aspirates and oral wash samples, and is a possible source of heterogeneity. Second, the sensitivity of the reference test is suboptimal, especially in non-HIV patients, as diagnostic accuracy of microscopy is highly dependent on fungal burden, type of microscope and stain, operator skill, experience, and time spent reading each slide, and this may have led to misclassification of cases. Even in papers that utilized IFA microscopy, which typically has higher sensitivity than conventional microscopy, the risk of misclassification remains, generally compromising the specificity of PCP PCR. Many of the studies reported several false-positive PCP PCR samples from patients where there was a strong clinical suspicion of PCP and positive response to treatment but negative microscopy, particularly after the initiation of empirical PCP treatment [22–31]. The heterogeneity of sample collection techniques within specimen types such as sample volume and quality, and operator skill when performing bronchoscopy could not be considered in the analyses due to lack of information. Finally, reporting was variable across studies. HIV status was not specified in nearly 30% of patients, which may have limited the power to assess the diagnostic performance of URT samples (for which there were comparatively fewer samples) according to HIV status. The sensitivity of conventional PCR was unexpectedly comparable to that of qPCR. The date of publication of included studies spanned close to 3 decades. The popularization of the 2 types of PCR in different historical ages may have led to significant heterogeneity. Over the last 3 decades, there have been changes in the prevalence of HIV among PCP cases, in the role of standard of care, and pressure to support the PCR as the main laboratory investigation, and selective reporting and publication bias may have overestimated the diagnostic accuracy of cPCR.

## CONCLUSIONS

This meta-analysis concluded that qPCR on BALF samples offered the highest sensitivity, while qPCR on IS also provided high sensitivity. There was no significant difference in sensitivity and specificity of PCR according to HIV status. When testing deeper respiratory specimens, PCR negativity can be used to confidently exclude PCP, while PCR positivity will likely require clinical interpretation, partially dependent on the strength of the PCR signal (indicative of fungal burden), specimen type, and patient population tested. The results provide a framework for clinicians on the interpretation of testing results using different specimens and PCR approaches.

## Supplementary Data

Supplementary materials are available at *Clinical Infectious Diseases* online. Consisting of data provided by the authors to benefit the reader, the posted materials are not copyedited and are the sole responsibility of the authors, so questions or comments should be addressed to the corresponding author.

## Supplementary Material

ciae239_Supplementary_Data

## Notes

***Author Contributions.*** Study conception and design: R. R.-R., P. L. W., M. C., J. P. D., and J. L. Data curation and formal analysis: L. B., M. C., R. R.-R., C. M., A. A., R. A. B., S. B., S. C.-A. A., C. C., J. P. D., W. J. H., B. J., L. K., J. L., T. R. R., E. R., P. L. W., and M. C. Methodology: L. B., M. C., C. M., J. P. D., R. R.-R., and P. L. W. Project administration: L. B., M. C., E. R., R. R.-R. Supervision: C. M., J. P. D., R. R.-R., P. L. W., and J. L. Visualization: C. M. and M. C. Writing–original draft: L. B., M. C., and C. M. Writing–review & editing: All authors.

***Acknowledgments.*** The authors thank Bethan Morgan and the Library Services at Wythenshawe Hospital, Manchester University National Health Service Foundation Trust, for assisting with the literature search.

***Data sharing.*** Data in this systematic review with meta-analysis were extracted from published and preprint studies available on the internet. Request for the meta-analysis data can be made by contacting the corresponding author.

***Financial support*.** This work was co-funded by the National Institute for Health and Care Research Manchester Biomedical Research Centre (NIHR203308).
