# Supplementary material for: Polymerase Chain Reaction on Respiratory Tract Specimens of Immunocompromised Patients to Diagnose Pneumocystis Pneumonia: A Systematic Review and Meta-analysis
Source: Clin Infect Dis. 2024 Jun 11;79(1):161–8. doi: 10.1093/cid/ciae239 (PMC11259226; doi:10.1093/cid/ciae239)
Supplement: ciae239_Supplementary_Data [file ciae239_supplementary_data.docx]

**Supplementary appendix**

**Title:** PCR on respiratory tract specimens of immunocompromised patients to diagnose Pneumocystosis (PcP): A systematic review and meta-analysis

**Authors**

Lottie Brown^1^, Riina Rautemaa-Richardson^2^, Carlo Mengoli^3^, Alexandre Alanio^4^, Rosemary A. Barnes^5^, Stéphane Bretagne^6^, Sharon C-A. Chen^7^, Catherine Cordonnier^8^, J. Peter Donnelly^9^, Werner J. Heinz^10^, Brian Jones^11^, Lena Klingspor^12^, Juergen Loeffler^13^, Thomas R Rogers^14^, Eleanor Rowbotham^15^, P. Lewis White^16^, Mario Cruciani^17^

**Affiliations**

1. LB: St George’s University and St George’s Hospital, London, UK; ORCID iD: 0000-0001-9896-8682.
2. RRR: Mycology Reference Centre Manchester and Department of Infectious Diseases, Manchester University, Manchester University NHS Foundation Trust, Wythenshawe Hospital, Manchester, UK; ORCID iD: 0000-0002-1071-6040
3. CM: Instituto Superiore Di Sanita, Rome, Italy; ORCID iD: 0000-0002-7422-0554
4. AA: Institut Pasteur, Unité de Mycologie Moléculaire, Paris, France; ORCID iD: [0000-0001-9726-3082](https://orcid.org/0000-0001-9726-3082)
5. RB: School of Medicine, University of Cardiff, UK; ORCID iD; 0000-0002-0574-8670
6. SB: Université Paris Cité, Paris, France; ORCID iD: [0000-0001-6870-3800](https://courriel-bsr.aphp.fr/owa/redir.aspx?C=CVdTZSoeJ0-J3oYFCrFhvAvmReVfgdMI1ThWHaXuE2dhiyesXlVp3yEoviZgXd5yMfKzmmGWKVA.&URL=http%3a%2f%2forcid.org%2f0000-0001-6870-3800)
7. SC: Centre for Infectious Diseases and Microbiology Laboratory Services, Institute of Clinical Pathology and Medical Research, New South Wales Health Pathology, Westmead Hospital, Westmead, New South Wales, Australia; ORCID iD: 0000-0002-9983-6603
8. CC: Haematology and stem cell transplant department, Henri Mondor hospital, and University Paris-Est-Créteil, Créteil, France
9. PD: Fungal PCR Initiative, a working group of the International Society of Human and Animal Mycology (ISHAM) Verona, Italy; ORCID iD: 0000-0002-1641-7367
10. WJH: Med. Clinic II, Caritas Hospital Bad Mergentheim, Germany; ORCID iD: 0000-0002-7833-9202
11. BJ: Institute of Infection Immunity and Inflammation, University of Glasgow, Glasgow, Scotland; ORCID iD: 0000-0002-9439-2353
12. LK: Karolinska Institutet, Department of Laboratory Medicine, Karolinska University Hospital Huddinge, Stockholm, Sweden; ORCID iD: 0000- 0001-8259-3556
13. JL: Universitätsklinikum Würzburg, Medizinische Klinik II, Labor WÜ4i, Würzburg, Germany
14. TR: Discipline of Clinical Microbiology, Trinity College Dublin, St James’s Hospital Campus, Dublin 8, Ireland; ORCID iD: 0000-0003-4336-7729
15. ER: Mycology Reference Centre Manchester and Department of Infectious Diseases, Manchester University, Manchester University NHS Foundation Trust, Wythenshawe Hospital, Manchester, UK
16. LW: Public Health Wales Mycology reference laboratory, PHW Microbiology Cardiff, UHW, and Centre for Trials Research/Division of Infection and Immunity, Cardiff University, Cardiff, UK; ORCID iD: 0000-0003-3056-4205
17. MC: Fungal PCR Initiative, a working group of the International Society of Human and Animal Mycology (ISHAM) Verona, Italy; ORCID iD: 0000-0002-1641-7367

**Key Words:** *Pneumocystis* PCR, Pneumocystosis, PcP, meta-analysis, systematic review

**Supplementary table 1: Summary of results of previous meta-analyses on PCR diagnosis of PcP and their limitations**

| **Author, year** | **Sensitivity (95% CI)** | **Specificity (95% CI)** | **Limitations** |
| --- | --- | --- | --- |
| Lu et al, 2011^12^ | Overall: 99% (96-100%)  BAL: 99% (99-100%)  IS: 97% (92-100%) | Overall: 90% (87-93%)  BAL: 87% (83-91%)  IS: 93% (90-95%) | - Only 13 included studies. - No analysis of URT samples. - No risk of bias assessment. |
| Fan et al, 2013^13^ | BAL: 98% (91–100%) | 91% (83–96%) | - Only 16 included studies. - BAL samples only. |
| Summah et al 2013^14^ | Overall: 97% (93-99%)  BAL: 98% (94-99%) | Overall: 94% (90-96%)  BAL: 93% (89-96%) | - Only 10 included studies – 2330 samples. - Subgroup analysis for BAL only. - PCR compared to both proven and probable PcP. |
| Senécal et al 2022^15^ | NPA: 89% (80-96%)  OW: 77% (66-85%)  IS: 99% (51-100%) | NPA: 98% (93-100%)  OW: 94% (90-96%)  IS: 96% (88-99%) | - Non-invasive samples only i.e. no BAL - Only 7 studies on IS PCR and, 13 URT PCR - All types of PCR pooled together - Inclusion of case-control studies. |

95% CI; 95% confidence interval, BAL; bronchoalveolar lavage, IS; induced sputum, URT; upper respiratory tract, NPA; nasopharyngeal aspirate, OW; oral wash, PCR; polymerase chain reaction; PcP; *Pneumocystis* pneumonia

**Supplementary 2: Search strategy**

Search Strategy: 1 exp Pneumocystosis/ 2 exp Pneumocystis pneumonias/ 3 exp Pneumocystis carinii Pneumonia/ Pneumocystis jirovecii pneumonia/ / PCP/ PJP 4 (Pneumocystis or Pneumocystosis or "P. jirovecii" or "P. carinii" ).ti,ab.5 or/1-4 6 exp Polymerase Chain Reaction/ 7 pcr.ti,ab. 8 "polymerase chain reaction*".ti,ab. 9 or/6-8 10 5 and 911 exp Animals/ not Humans/12 10 not 11.

^1-54^

**Supplementary 3: References of articles included in the meta-analysis**

1. Alanio A, Desoubeaux G, Sarfati C, et al. Real-time PCR assay-based strategy for differentiation between active Pneumocystis jirovecii pneumonia and colonization in immunocompromised patients. *Clinical microbiology and infection* 2011; **17**(10): 1531-7.

2. Alshahrani MY, Alfaifi M, Ahmad I, et al. Pneumocystis Jirovecii detection and comparison of multiple diagnostic methods with quantitative real-time PCR in patients with respiratory symptoms. *Saudi journal of biological sciences* 2020; **27**(6): 1423-7.

3. Azoulay E, Bergeron A, Chevret S, Bele N, Schlemmer B, Menotti J. Polymerase chain reaction for diagnosing pneumocystis pneumonia in non-HIV immunocompromised patients with pulmonary infiltrates. *Chest* 2009; **135**(3): 655-61.

4. Caliendo AM, Hewitt PL, Allega JM, Keen A, Ruoff KL, Ferraro MJ. Performance of a PCR assay for detection of Pneumocystis carinii from respiratory specimens. *Journal of Clinical Microbiology* 1998; **36**(4): 979-82.

5. Cartwright CP, Nelson NA, Gill VJ. Development and evaluation of a rapid and simple procedure for detection of Pneumocystis carinii by PCR. *Journal of Clinical Microbiology* 1994; **32**(7): 1634-8.

6. Chotiprasitsakul D, Pewloungsawat P, Setthaudom C, Santanirand P, Pornsuriyasak P. Performance of real-time PCR and immunofluorescence assay for diagnosis of Pneumocystis pneumonia in real-world clinical practice. *Plos one* 2020; **15**(12): e0244023.

7. Chawla K, Martena S, Gurung B, Mukhopadhyay C, Varghese GK, Bairy I. Role of PCR for diagnosing Pneumocystis jirovecii pneumonia in HIV-infected individuals in a tertiary care hospital in India. *Indian J Pathol Microbiol* 2011; **54**(2): 326-9.

8. Chouaid C, Roux P, Lavard I, Poirot JL, Housset B. Use of the polymerase chain reaction technique on induced-sputum samples for the diagnosis of Pneumocystis carinii pneumonia in HIV-infected patients: a clinical and cost-analysis study. *American journal of clinical pathology* 1995; **104**(1): 72-5.

9. Chumpitazi BF, Flori P, Kern J-B, et al. Characteristics and clinical relevance of the quantitative touch-down major surface glycoprotein polymerase chain reaction in the diagnosis of Pneumocystis pneumonia. *Medical mycology* 2011; **49**(7): 704-13.

10. Church DL, Ambasta A, Wilmer A, et al. Development and validation of a Pneumocystis jirovecii real-time polymerase chain reaction assay for diagnosis of Pneumocystis pneumonia. *Canadian Journal of Infectious Diseases and Medical Microbiology* 2015; **26**(5): 263-7.

11. Desoubeaux G, Franck-Martel C, Caille A, et al. Use of calcofluor-blue brightener for the diagnosis of Pneumocystis jirovecii pneumonia in bronchial-alveolar lavage fluids: A single-center prospective study. *Medical Mycology* 2017; **55**(3): 295-301.

12. Eisen D, Ross BC, Fairbairn J, Warren RJ, Baird RW, Dwyer B. Comparison of Pneumocystis carinii detection by toluidine blue O staining, direct immunofluorescence and DNA amplification in sputum specimens from HIV positive patients. *Pathology* 1994; **26**(2): 198-200.

13. Evans R, Joss A, Parratt D, Pennington T, Ho-Yen D. The role of a nested polymerase chain reaction in the diagnosis of Pneumocystis carinii pneumonia. *Clinical Molecular Pathology* 1995; **48**(6): M347.

14. Evans R, Joss A, Pennington T, Ho-Yen D. The use of a nested polymerase chain reaction for detecting Pneumocystis carinii from lung and blood in rat and human infection. *Journal of medical microbiology* 1995; **42**(3): 209-13.

15. Fillaux J, Malvy S, Alvarez M, et al. Accuracy of a routine real-time PCR assay for the diagnosis of Pneumocystis jirovecii pneumonia. *Journal of Microbiological Methods* 2008; **75**(2): 258-61.

16. Fischer S, Gill VJ, Kovacs J, et al. The use of oral washes to diagnose Pneumocystis carinii pneumonia: a blinded prospective study using a polymerase chain reaction–based detection system. *The Journal of infectious diseases* 2001; **184**(11): 1485-8.

17. Goterris L, Mancebo Fernández MA, Aguilar-Company J, Falcó V, Ruiz-Camps I, Martín-Gómez MT. Molecular diagnosis of pneumocystis jirovecii pneumonia by use of oral wash samples in immunocompromised patients: usefulness and importance of the DNA target. *Journal of clinical microbiology* 2019; **57**(12): e01287-19.

18. Graves DC, Chary-Reddy S, Becker-Hapak M. Detection ofPneumocystis cariniiin induced sputa from immunocompromised patients using a repetitive DNA probe. *Molecular and cellular probes* 1997; **11**(1): 1-9.

19. Hauser PM, Bille J, Lass-Flörl C, et al. Multicenter, prospective clinical evaluation of respiratory samples from subjects at risk for Pneumocystis jirovecii infection by use of a commercial real-time PCR assay. *Journal of clinical microbiology* 2011; **49**(5): 1872-8.

20. Helweg-Larsen J, Skov Jensen J, Benfield T, Gerner Svendsen U, Lundgren JD, Lundgren B. Diagnostic use of PCR for detection of Pneumocystis carinii in oral wash samples. *Journal of clinical microbiology* 1998; **36**(7): 2068-72.

21. Hoarau G, Le Gal S, Zunic P, et al. Evaluation of quantitative FTD-Pneumocystis jirovecii kit for Pneumocystis infection diagnosis. *Diagnostic Microbiology and Infectious Disease* 2017; **89**(3): 212-7.

22. Huang SN, Fischer SH, O’Shaughnessy E, Gill VJ, Masur H, Kovacs JA. Development of a PCR assay for diagnosis of Pneumocystis carinii pneumonia based on amplification of the multicopy major surface glycoprotein gene family. *Diagnostic microbiology and infectious disease* 1999; **35**(1): 27-32.

23. Huggett JF, Taylor MS, Kocjan G, et al. Development and evaluation of a real-time PCR assay for detection of Pneumocystis jirovecii DNA in bronchoalveolar lavage fluid of HIV-infected patients. *Thorax* 2008; **63**(2): 154-9.

24. Juliano JJ, Barnett E, Parobek CM, et al. Use of Oropharyngeal Washes to Diagnose and Genotype Pneumocystis jirovecii. *Open Forum Infect Dis* 2015; **2**(3): ofv080.

25. Kaouech E, Kallel K, Anane S, et al. Pnemocystis jiroveci pneumonia: comparison between conventional PCR and staining techniques. *Pathologie-biologie* 2008; **57**(5): 373-7.

26. Kilic A, Elliott S, Hester L, Palavecino E. Evaluation of the performance of DiaSorin molecular Pneumocystis jirovecii-CMV multiplex real-time PCR assay from bronchoalveolar lavage samples. *Journal de mycologie medicale* 2020; **30**(2): 100936.

27. Larsen HH, Huang L, Kovacs JA, et al. A prospective, blinded study of quantitative touch-down polymerase chain reaction using oral-wash samples for diagnosis of Pneumocystis pneumonia in HIV-infected patients. *The Journal of infectious diseases* 2004; **189**(9): 1679-83.

28. Lipschik GY, Andrawis VA, Ognibene FP, et al. Improved diagnosis of Pneumocystis carinii infection by polymerase chain reaction on induced sputum and blood. *The Lancet* 1992; **340**: 203-6.

29. Mathis A, Weber R, Kuster H, Speich R. Simplified sample processing combined with a sensitive one-tube nested PCR assay for detection of Pneumocystis carinii in respiratory specimens. *Journal of Clinical Microbiology* 1997; **35**(7): 1691-5.

30. Bautista MM, Qi C. Comparative Evaluation between Real-Time PCR Reaction Assay and Direct Immunofluorescence Antibody for Pneumocystis jirovecii. LABORATORY INVESTIGATION; 2020: NATURE PUBLISHING GROUP 75 VARICK ST, 9TH FLR, NEW YORK, NY 10013-1917 USA; 2020. p. 1425-6.

31. Moodley B, Tempia S, Frean JA. Comparison of quantitative real-time PCR and direct immunofluorescence for the detection of Pneumocystis jirovecii. *PLoS One* 2017; **12**(7): e0180589.

32. Matsumura Y, Ito Y, Iinuma Y, et al. Quantitative real-time PCR and the (1→ 3)-β-D-glucan assay for differentiation between Pneumocystis jirovecii pneumonia and colonization. *Clinical microbiology and infection* 2012; **18**(6): 591-7.

33. Moonens F, Liesnard C, Brancart F, Van Vooren JP, Serruys E. Rapid simple and nested polymerase chain reaction for the diagnosis of Pneumocystis carinii pneumonia. *Scandinavian journal of infectious diseases* 1995; **27**(4): 358-62.

34. Rabodonirina M, Raffenot D, Cotte L, et al. Rapid detection of Pneumocystis carinii in bronchoalveolar lavage specimens from human immunodeficiency virus-infected patients: use of a simple DNA extraction procedure and nested PCR. *Journal of clinical microbiology* 1997; **35**(11): 2748-51.

35. Robberts FJ, Liebowitz LD, Chalkley LJ. Polymerase chain reaction detection of Pneumocystis jiroveci: evaluation of 9 assays. *Diagnostic microbiology and infectious disease* 2007; **58**(4): 385-92.

36. Robert-Gangneux F, Belaz S, Revest M, et al. Diagnosis of Pneumocystis jirovecii pneumonia in immunocompromised patients by real-time PCR: a 4-year prospective study. *Journal of clinical microbiology* 2014; **52**(9): 3370-6.

37. Roux P, Lavrard I, Poirot J, et al. Usefulness of PCR for detection of Pneumocystis carinii DNA. *Journal of Clinical Microbiology* 1994; **32**(9): 2324-6.

38. Samuel CM, Whitelaw A, Corcoran C, et al. Improved detection of Pneumocystis jirovecii in upper and lower respiratory tract specimens from children with suspected pneumocystis pneumonia using real-time PCR: a prospective study. *BMC infectious diseases* 2011; **11**(1): 1-6.

39. Sasso M, Chastang-Dumas E, Bastide S, et al. Performances of four real-time PCR assays for diagnosis of Pneumocystis jirovecii pneumonia. *Journal of clinical microbiology* 2016; **54**(3): 625-30.

40. Savoia D, Millesimo M, Cassetta I, Forno B, Caramello P. Detection of Pneumocystis carinii by DNA amplification in human immunodeficiency virus-positive patients. *Diagnostic microbiology and infectious disease* 1997; **29**(2): 61-5.

41. Sing A, Trebesius K, Roggenkamp A, et al. Evaluation of diagnostic value and epidemiological implications of PCR for Pneumocystis carinii in different immunosuppressed and immunocompetent patient groups. *Journal of Clinical Microbiology* 2000; **38**(4): 1461-7.

42. Singh P, Singh S, Mirdha BR, Guleria R, Agarwal SK, Mohan A. Evaluation of loop-mediated isothermal amplification assay for the detection of Pneumocystis jirovecii in immunocompromised patients. *Molecular biology international* 2015; **2015**.

43. Takahashi T, Goto M, Endo T, et al. Pneumocystis carinii carriage in immunocompromised patients with and without human immunodeficiency virus infection. *Journal of medical microbiology* 2002; **51**(7): 611-92.

44. Tamburrini E, Mencarini P, Visconti E, et al. Imbalance between Pneumocystis carinii cysts and trophozoites in bronchoalveolar lavage fluid from patients with pneumocystosis receiving prophylaxis. *Journal of medical microbiology* 1996; **45**(2): 146-8.

45. To KK, Wong SC, Xu T, et al. Use of nasopharyngeal aspirate for diagnosis of pneumocystis pneumonia. *Journal of clinical microbiology* 2013; **51**(5): 1570-4.

46. Torres J, Goldman M, Wheat LJ, et al. Diagnosis of Pneumocystis carinii Pneumonia in Human Immunodeficiency Virus—Infected Patients with Polymerase Chain Reaction: A Blinded Comparison to Standard Methods. *Clinical infectious diseases* 2000; **30**(1): 141-5.

47. Wakefield A, Guiver L, Miller R, Hopkin J. DNA amplification on induced sputum samples for diagnosis of Pneumocystis carinii pneumonia. *The Lancet* 1991; **337**(8754): 1378-9.

48. Wang D, Hu Y, Li T, Rong H-M, Tong Z-H. Diagnosis of Pneumocystis jirovecii pneumonia with serum cell-free DNA in non-HIV-infected immunocompromised patients. *Oncotarget* 2017; **8**(42): 71946.

49. Zingale A, Carrera P, Lazzarin A, Scarpellini P. Detection of Pneumocystis carinii and characterization of mutations associated with sulfa resistance in bronchoalveolar lavage samples from human immunodeficiency virus-infected subjects. *Journal of clinical microbiology* 2003; **41**(6): 2709-12.

50. Almeida-Siva F, Almeida-Paes R, Serra-Damasceno L, et al. The conventional diagnosis challenge: Real-time PCR and nested PCR correlation with the scoring system for individuals at high-risk of Pneumocystis jirovecii pneumonia. *Biomedica* 2023; **43**(Sp. 1): 255-66.

51. Barbin NJ, Tappeh KH, Diba K, Makhdoumi K, Khademvatan S. Comparison Of Current Diagnostic Methods And Molecular Identification Of Pneumocystis Jirovecii Causing PJP In Cases Immune Suppressed Patients At The Nephrology Ward, Imam Khomeini Hospital, Urmia. *Journal of Pharmaceutical Negative Results* 2022: 1953-66.

52. Franconi I, Leonildi A, Erra G, et al. Comparison of different microbiological procedures for the diagnosis of Pneumocystis jirovecii pneumonia on bronchoalveolar-lavage fluid. *BMC microbiology* 2022; **22**(1): 1-5.

53. Salsé M, Mercier V, Carles Mj, Lechiche C, Sasso M. Performance of the RealStar® Pneumocystis jirovecii PCR kit for the diagnosis of Pneumocystis pneumonia. *Mycoses* 2021; **64**(10): 1230-7.

54. Veintimilla C, Álvarez-Uría A, Martín-Rabadán P, et al. Pneumocystis jirovecii Pneumonia Diagnostic Approach: Real-Life Experience in a Tertiary Centre. *Journal of Fungi* 2023; **9**(4): 414.

**Supplementary 4: Study characteristics**

| **Study (n=55)** | **Country** | **Centres** | **Patients (n)** | **Patient population** |
| --- | --- | --- | --- | --- |
|  |  |  |  |  |
| Alanio 2011 | France | 1 | 238 | Adults, HIV (69) and non HIV (169) including haemotological malignancy, solid organ transplant, solid malignancy and autoimmune on immunosuppressants |
| Almeida-Silva 2023 | Brazil | 1 | 40 | Adult patients (40), all with HIV and CD4<200 |
| Alshahrani 2020 | Saudi Arabia | "Multi" | 100 | Adults, form of immunocompromise not specified |
| Azoulay 2009 | France | 1 | 448 | Age range not specified, all non-HIV including haematological malignancy, solid malignancy, solid organ and stem cell transplant, autoimmune on immunosuppressants |
| Barbin 2022 | Iran | 7 | 200 | Age range not specified. All 200 hospitalized patients receiving prolonged immunosuppressive therapies (kidney transplant recipients, patients with autoimmune disorders and malignant disorders under chemotherapy) |
| Caliendo 1998 | USA | 1 | 168 | Age range not specified, HIV and non-HIV including solid organ transplant recipients |
| Cartwright 1994 | USA | 1 | 205 | Age range not specified, HIV (112), non-HIV including malignancies (73) and other causes of immunocompromise not specified |
| Chawla 2011 | India | 1 | 50 | Adults with HIV |
| Chotiprasitsakul 2020 | Thailand | 1 | 222 | Adults with HIV (37) and non-HIV (185), including haemotological malignancy, solid organ transplant, solid malignancy and autoimmune on immunosuppressants |
| Chouaid 1995 | France | 1 | 49 | Adults with HIV |
| Chumpitazi 2011 | France | 1 | 54 | Adults and children, with HIV (5) and non-HIV (49) including haemotological malignancy, solid organ transplant, solid malignancy and autoimmune on immunosuppressants |
| Church 2015a | Canada | 1 | 127 | Age range not specified, HIV (9) and non-HIV (78) including malignancy, solid organ and stem cell transplant recipients |
| Desoubeaux 2017 | France | 1 | 481 | Adults with HIV (6) and non-HIV (42) including haemotological malignancy, solid organ transplant, solid malignancy and inherited immunodeficiencies |
| Eisen 1994 | Australlia | 1 | 20 | Age range not specified, all with HIV |
| Evans 1995a | UK | 1 | NS | Age range or immunocompromise not specified |
| Evans 1996b | UK | 3 | 53 | Age range not specified, HIV (11) and non-HIV (42) with other forms of immunocompromise non-specified |
| Fillaux 2008 | France | 1 | 101 | Adults and children, HIV (22) and non-HIV, malignancy (36) and other non-specified |
| Fischer 2001 | USA | 1 | 175 | Age range or immunocompromise not specified |
| Franconi 2022 | Italy | 1 | 18 | Age range not specified. Both HIV and non-HIV patients included all on immunosuppressive therapies. |
| Goterris 2019 | Spain | 1 | 36 | Age range not specified, HIV (5) and non-HIV (31) including haemotological malignancy, solid organ transplant, solid malignancy and autoimmune on immunosuppressants |
| Graves 1997 | USA | 1 | 74 | Age range not specified, HIV (52) and non-HIV (22) not specified |
| Hauser 2011 | UK, Austria, Switzerland, USA | 6 | 110 | Adults with HIV (9) and non-HIV (101) including solid organ transplant and malignancy |
| Helweg 1998 | Denmark | 1 | 76 | Age range not specified, all with HIV |
| Hoarau 2017 | Reunion | 1 | 133 | Age range not specified, HIV (13) and non-HIV (120) including haemotological malignancy, solid organ transplant, solid malignancy |
| Huang 1999 | USA | 1 | NS | Age range and immunocompromise not specified |
| Huggett 2009 | UK | NS | 61 | Age range not specified, all with HIV |
| Juliano 2015 | USA | 1 | 43 | Adults with HIV |
| Kaouech 2008 | Tunisia | NS | 54 | Children and adults, HIV (20) and non-HIV (34) including haemotological malignancy, cirrhosis, autoimmune, immunodeficiency |
| Kilic 2020 | USA | 1 | 125 | Age range not specified, HIV (35) and non-HIV (90) including malignancies, stem cell or bone marrow transplant, solid organ transplant and other immunosuppressed condtions |
| Larsen 2004 | USA | 1 | 108 | Age range not specified, all with HIV |
| Lipschik 1992 | USA | NS | 133 | Age range not specified, HIV (65) and non-HIV (68) including maligancy, autoimmune and other not specified |
| Mathis 1997 | Switzerland | 1 | 507 | Age range not specified, HIV (295) and non-HIV (164) including organ transplant, haemotological malignancy, immunosuppressants |
| Matsumura 2012 | Japan | 1 | 147 | Age range not specified, HIV (13) and non-HIV (134) including solid and haemotological malignancy, autoimmune, organ transplant |
| Mejia Bautista 2020 | USA | 1 | NS | Age range and immunocompromise not specified |
| Moodley 2017 | South Africa | 1 | 266 | Adults with HIV |
| Moonens 1995 | Belgium | 1 | 43 | Age range not specified, HIV (14) and non-HIV (29) including solid organ transplant recipients and immunosuppressants |
| Rhabodonirina 1997 | France | 1 | 105 | Age range not specified, all with HIV |
| Robberts 2007 | South Africa | 1 | NS | Age range and immunocompromise not specified |
| Robert-Gangneux 2014 | France | 1 | 659 | Age range not specified, HIV and non-HIV including haemotological malignancy, solid malignancy, transplant recipients and immunosuppressants |
| Roux 1994 | France | 1 | 165 | Age not specified, HIV (120) and non-HIV (45) not specified |
| Salsé 2021 | France | 1 | 219 | Adult patients, HIV (12) and non-HIV (207) including haematological malignancy, solid tumours and autoimmune or inflammatory disease |
| Samuel 2011 | South Africa | 1 | 202 | Children with HIV (129) and non-HIV (71) including malnutrition, receiving immunosuppressive therapy or immunodeficiency disease other than HIV |
| Savoia 1997 | Italy | 1 | 123 | Age range not specified, all with HIV |
| Sasso 2016 | France | 1 | 148 | Adults and children with HIV (40) and non-HIV (108) including solid malignancy, haemotological malignancy, solid organ and stem cell transplant, autoimmune and underlying respiratory disease |
| Sing 2000 | Germany | 1 | 375 | Adults and children with HIV (89) and non-HIV (245) including transplant recipients, malignancy and other non-specified |
| Singh 2015 | India | 1 | 185 | Adults with HIV (64) and non-HIV (121) including renal transplant recipients, malignancy, and other non-specified |
| Takahashi 2002 | Japan | 1 | 81 | Adults and children, HIV (26) and non-HIV (55) including haemotological malignancy, connective tissue diseases and renal transplantation |
| Tamburrini 1993 | Italy | 1 | 52 | Adults with HIV (48) and non-HIV (4) |
| Tia 2012 | Thailand | 1 | 102 | Age range not specified, HIV (66) and non-HIV (36) including underlying solid organ malignancies, haematological malignancies, systemic lupus erythematosus and organ transplantation |
| To 2013 | Hong Kong | "Multi" | 117 | Age range not specified, HIV (10) and non-HIV (107) including lung disease, malignancy, stem cell or solid organ transplant recipient, autoimmune on immunosuppressants |
| Torres 2000 | USA | 2 | 47 | Adults with HIV |
| Veintimilla 2023 | Spain | 1 | 299 | Adults, both HIV (53) and. Non-HIV (246) including patients with haematological malignancy, solid tumours, transplant recipients, on immunosuppressants |
| Wakefield 1991 | UK | 1 | 47 | Age range not specified, all with HIV |
| Wang 2017 | China | 1 | 71 | Age range not specified, non-HIV including solid organ transplant, malignancy, autoimmune condition on immunosuppressants |
| Zingale 2003 | Italy | 1 | 173 | Adults with HIV |

NS; not specified

**Supplementary 5: PCR techniques**

| **Study (n=55)** | **Type of microscopy in reference standard** | **PCR** | **DNA extraction** | **Sample volume for DNA extraction (μL)** | **Target gene** | **Internal control** |
| --- | --- | --- | --- | --- | --- | --- |
| Alanio 2011 | High probability': Giemsa stain + indirect IF +/- direct IF (if prev were neg). Decided by 2 blinded clinicians. | In-house qPCR | QIAamp DNA mini kit | 200 | mtLSU rRNA | Yes |
| Almieda 2023 | IFA | In-house nested cPCR/Commerical qPCR (Viasure) | QIAamp DNA Mini kit | NS | mtLSU rRNA |  |
| Alsharani 2020 | Clinically suspected': Direct IF | Commerical qPCR (MycAssay) | QIAamp DNA mini kit | NS | mtLSU rRNA | Yes |
| Azoulay 2009 | Definitive': Giemsa + indirect IFA (2x slides if prev neg and PCR pos). 'Probable': negative microbiology, response to treatment | In-house cPCR | QIAamp DNA mini kit | NS | LSU rRNA | Yes |
| Barbin 2022 | GMS and giemsa | In-house cPCR | phenol chloroform | NS | mtLSU rRNA |  |
| Caliendo 1998 | Direct IFA | Commercial colorimetric cPCR (Roche) | Organic | 100 | 18s rRNA | Yes |
| Cartwright 1994 | Direct IFA | In-house PCR-EIA (ELISA) | Organic | 500-2000 | mtLSU rRNA | No |
| Chawla 2011 | GMS and giemsa. | In-house cPCR | QIAamp DNA mini kit | NS | mtLSU rRNA | Yes |
| Chotiprasitsaku 2020 | Direct IFA | Commerical qPCR (Biopharm AG) | magLEAD | NS | mtLSU rRNA | Yes |
| Chouaid 1995 | Giemsa, Musto, GMS. IS also by direct IFA. | In-house cPCR | Phenolchloroform | NS | mtLSU rRNA | Yes |
| Chumpitazi 2011 | Confirmed': Giemsa, GMS | In-house qPCR touch-down | Qiagen EZ1 DNA tissue kit | 400 | MSG | Yes |
| Church 2015 | Modified gold standard': TDO, GMS OR direct IFA OR if neg by IFA + pos x2 by PCR, considered positive. Also made comparison to IFA only. | In-house qPCR | Quickgene 810 | 180 | mtLSU rRNA | Yes |
| Desoubeaux 2017 | Direct IFA | In-house qPCR | QIAamp DNA mini kit | 200 | mtLSU rRNA | Yes |
| Eisen 1994 | Direct IFA | In-house cPCR | Organic | 500 | mtLSU rRNA | Yes |
| Evans 1995a | Direct IFA | In-house nested cPCR | Phenolchloroform | NS | mtLSU rRNA | Yes |
| Evans 1996b | Direct IFA | In-house cPCR | Phenolchloroform | 20 | mtLSU rRNA | Yes |
| Fillaux 2008 | Direct IFA | In-house qPCR | Roche, isopropanol-based | 1500 | MSG | Yes |
| Fischer 2001 | Direct IFA or GMS (depending on centre) | In-house immunofluorescent cPCR | InstaGene Matrix (Bio-Rad) | 5000-50,000 | MSG/mtLSU | Yes |
| Franconi 2022 | GMS | Commercial qPCR (Unyvero/  Saccace) | Commerical Unyvero kit | 180 | 26s rRNA |  |
| Goterris 2019 | Direct IFA | qPCR | QIAamp DNA minikit | NS | DHPS/ mtSSU rRNA | Yes |
| Graves 1997 | Direct IFA | cPCR | Organic | 5000 | Dihydrofolate reductase gene | Yes |
| Hauser 2011 | Direct IFA | Commercial qPCR (MycAssay) | MycXtra fungal DNA extraction kit | <1000-60,000 | mtLSU rRNA | Yes |
| Helweg 1998 | Giemsa and direct IFA on BAL | In-house cPCR | Phenolchloroform | 2000 | mtLSU rRNA | Yes |
| Hoarav 2017 | Musto stain on BAL | Commercial qPCR (Fast Track Diagnostics) | NS | 600 | mtLSUrRNA | Yes |
| Huang 1999 | Direct IFA | In-house cPCR | DNA Extraction Reagent (Perkin Elmer) | IS (1000-3000), OW (10,000-15,000), BAL (5000-10,000) | MSG/mtLSU rRNA | Yes |
| Huggett 2009 | GMS | In-house qPCR (HSP70) and cPCR (MtLSU rRNA) | DNeasy tissue kit and subsequently the QIAamp UltraSens Virus Kit | 200 (DNeasy), 750 (Qiamp) | HSP70 DNA/ mtLSU rRNA | Yes |
| Juliano 2015 | Microscopy' non-specified on BAL/IS | In-house qPCR | QIAamp DNA mini kit | 15,000 | DHPS/MSG | Yes |
| Kaouech 2008 | Giemsa, GMS, TDO | In-house cPCR | QIAamp DNA Mini kit | NS | MSG | Yes |
| Kilic 2020 | Direct IFA | Commercial qPCR (DiaSorin Molecular) | Diasorin Molecular | NS | mtLSU rRNA | Yes |
| Larsen 2004 | Diff-quik on BAL/IS | In-house touch-down qPCR TD | NucliSens kit | NS | MSG | Yes |
| Lipschik 1992 | Indirect IFA, TDO | In-house cPCR (nested and simple) | Phenol chloroform | 5000-10,000 (BAL), 1000-3000 (IS) | mt rRNA | Yes |
| Mathis 1997 | Direct IFA | In-house cPCR | Biorad mesh | 1500 (BAL), 50-500 for other specimens | mtLSU rRNA | Yes |
| Matsumura 2012 | GMS | In-house qPCR | QIAamp DNA mini kit | NS | DHPS | Yes |
| Mejia 2020 | Direct IFA | In-house qPCR | Diasorin Molecular | NS | NS | NS |
| Moodley 2017 | Direct IFA | In-house qPCR | Roche MagNA Pure Compact | NS | mtLSU rRNA | Yes |
| Moonens 1995 | Direct IFA | In-house cPCR (nested and simple) | Phenolchloroform | 10,000 | mtLSU rRNA | Yes |
| Rhabodonirina 1997 | GMS and giemsa. | In-house cPCR (simple and nested) | GeneReleaser | NS | mtLSU rRNA | Yes |
| Robberts 2007 | Direct IFA | In-house nested cPCR | DNA purification kit, Wizard SV Genomic | 200-1000 | mtLSU rRNA | Yes |
| Robert-Gagneux 2014 | Giemsa and direct IFA on BAL | In-house qPCR | QIAamp DNA mini kit | 1000 | mtLSU rRNA | Yes |
| Roux 1994 | Giemsa, TDO or direct IFA | In-house cPCR | Phenolchloroform | 10,000 | mtLSU rRNA | NS |
| Salse 2021 | GMS and giemsa on BALF | Commercial qPCR (RealStar)/In-house qPCR/ | EZ1 DNA Tissue Kit (Qiagen) | 2000 | mtLSU rRNA/MSG |  |
| Samuel 2011 | Direct IFA | In-house qPCR | Nuclisens EasyMAG | NS | MSG | Yes |
| Saovia 1997 | Direct wet mount, Giemsa, TDO | In-house nested cPCR | Organic | NS | mtLSU rRNA | Yes |
| Sasso 2016 | Direct Giemsa, GG | In-house qPCR, commercial qPCRs (Amplisens, McAssay, Bioevolution) | EZ1 DNA Tissue kit (Qiagen®) | 2000 | MSG/ mtLSU rRNA | NS |
| Sing 2000 | Giemsa + GMS | In-house nested cPCR | Qiagen DNeasy tissue kit | NS | mtLSU rRNA | Yes |
| Singh 2015 | GMS | In-house cPCR (LAMP/nested) | Qiagen DNeasy tissue kit | 200 | mtLSU rRNA/18s rRNA | Yes |
| Takahashi 2002 | Diff-quik on BAL | In-house cPCR | Phenolchloroform | NS | 5S rDNA | Yes |
| Tamburrini 1993 | Direct IFA | In-house cPCR | Phenolchloroform | 20 | mtLSU rRNA | Yes |
| Tia 2012 | Giemsa and IFA on BAL | In-house cPCR (single round/nested) | QIAamp DNA mini kit | 1000 | mtLSU rRNA | Yes |
| To 2013 | GMS | In-house qPCR | QIAamp DNA kit and DNeasy plant minikit | 200 | mtSSU rRNA | Yes |
| Torres 2000 | GMS or Giemsa. | In-house cPCR (semi-quantitative +1 to +3) | NS | 5000-25,000 | ITS | NS |
| Veintimilla 2023 | Direct IFA (matched sample) | Commercial qPCR (Progenie Molecular) | MagnaPure Compact instrument using the Nucleic Acid Isolation kit I (Roche Diagnostics GmbH®), | 1500 | LSU rRNA |  |
| Wakefield 1991 | GMS and others. | In-house cPCR | Phenolchloroform | 1000 | LSU rRNA | NS |
| Wang 2017 | GMS | In-house qPCR | QIAamp DNA Mini kit | NS | HSP70 gene | NS |
| Zingale 2003 | Giemsa and Grocott | In-house qPCR | Qiagen tissue kit | NS | LSU rRNA/ DHPS | NS |

IFA; immunofluorescent assay, qPCR; quantitative PCR, GMS; Grocott methenamine silver, TBO; toluidine blue 0, NS; not specified,**Supplementary 6: Risk of bias and applicability concerns graph: review authors' judgements about each domain presented as percentages across included studies**


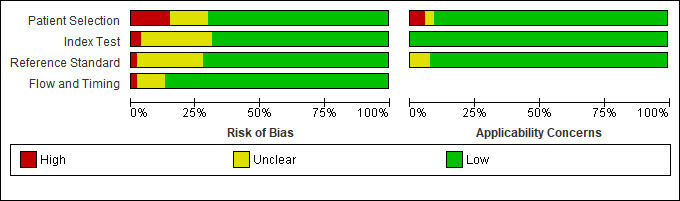


**Supplementary 7: Overall risk of bias and applicability concerns for 55 selected studies**


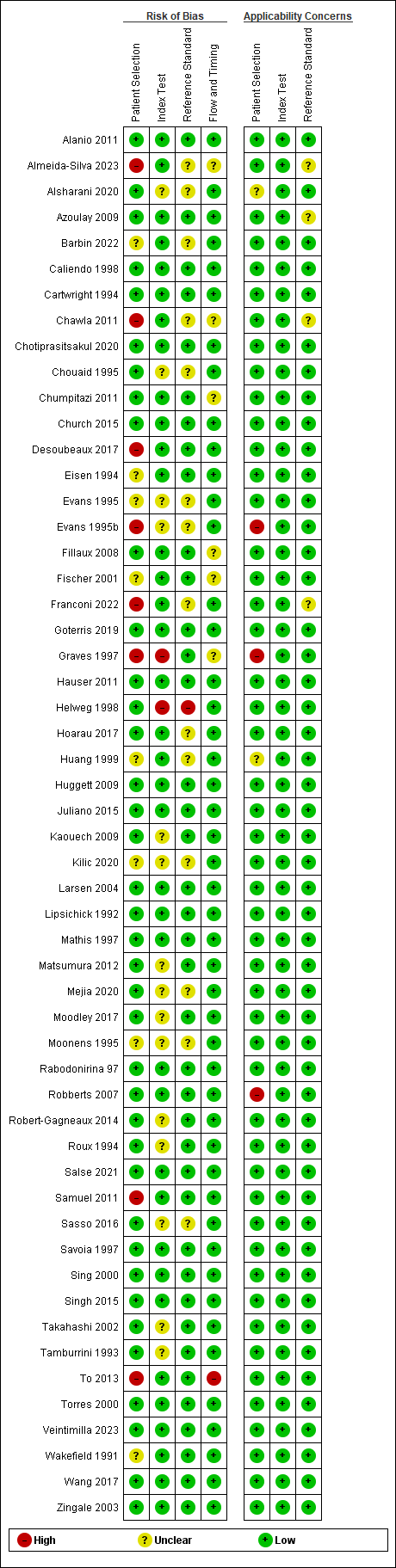
**Supplementary 8: Coefficients of the binomial regression**

| testpos | Coefficient | Std. Err | z | P | 95% Conf. Interval | |
| --- | --- | --- | --- | --- | --- | --- |
|  |  |  |  |  |  |  |
| 1.disease | 6.3783 | 0.4202 | 15.18 | 0.000 | 5.5546 | 7.2020 |
|  |  |  |  |  |  |  |
| sample |  |  |  |  |  |  |
| BALF and IS | 0.8159 | 0.4684 | 1.74 | 0.082 | -0.1021 | 1.7340 |
| IS | 0.6293 | 0.2253 | 2.79 | 0.005 | 0.1877 | 1.0708 |
| upper and lower | 0.3061 | 0.4224 | 0.72 | 0.469 | -0.5218 | 1.1340 |
| URT | -0.3457 | 0.4846 | -0.71 | 0.476 | -1.2955 | 0.6041 |
|  |  |  |  |  |  |  |
| disease#sample |  |  |  |  |  |  |
| 1#BALF and IS | -1.2122 | 0.7732 | -1.57 | 0.117 | -2.7278 | 0.3033 |
| 1#IS | -1.1679 | 0.4393 | -2.66 | 0.008 | -2.0290 | -0.3068 |
| 1#upper and lower | -0.5417 | 0.7111 | -0.76 | 0.446 | -1.9355 | 0.8522 |
| 1#URT | -2.1379 | 0.6340 | -3.37 | 0.001 | -3.3806 | -0.8952 |
|  |  |  |  |  |  |  |
| pcr |  |  |  |  |  |  |
| conventional | -0.8802 | 0.3017 | -2.92 | 0.004 | -1.4715 | -0.2888 |
|  |  |  |  |  |  |  |
| disease#pcr |  |  |  |  |  |  |
| 1#conventional | 0.1903 | 0.4801 | 0.4 | 0.692 | -0.7507 | 1.1313 |
|  |  |  |  |  |  |  |
| _cons | -2.1415 | 0.2386 | -8.97 | 0.000 | -2.6092 | -1.6738 |
|  |  |  |  |  |  |  |
| study |  |  |  |  |  |  |
| var(disease) | 1.0850 | 0.5367 |  |  | 0.4115 | 2.8608 |
| var(_cons) | 0.8637 | 0.2237 |  |  | 0.5199 | 1.4350 |
|  |  |  |  |  |  |  |
| study |  |  |  |  |  |  |
| cov(disease,_cons) | -0.0744 | 0.2442 | -0.3 | 0.761 | -0.5531 | 0.4043 |

Mixed-effects logistic regression. Dependent variable: “testpos”. Predictors: “disease”, “sample”, and “pcr”. Two interactions were included: “disease*sample” and “disease*pcr”. The group variable was “study”. The random effect intercept was “study”, the random coefficient was “disease”. Note that the baseline level for “sample” was “BALF”, for “pcr” was “qPCR”. Number of observations = 9,564. Number of groups = 49. Log likelihood = -2893.602. Wald chi2(11) = 457.44. Prob from chi2 = 0.0000. BALF; bronchoalveolar lavage fluid, IS; induced sputum, URT; upper respiratory tract. Std. Error; standard error, 95% CI; 95% confidence interval.

**Supplementary 9: Sensitivity, specificity, DOR, LR+ and LR- calculated from the coefficients of the binomial regression model.**

|  | **sens** | ll | ul | **spec** | ll | ul | **DOR** | ll | ul | **LR+** | ll | ul | **LR-** | ll | ul |
| --- | --- | --- | --- | --- | --- | --- | --- | --- | --- | --- | --- | --- | --- | --- | --- |
| qPCR, BALF | 0.987 | 0.968 | 0.995 | 0.893 | 0.844 | 0.927 | 635 | 269 | 1498 | 9.194 | 5.727 | 12.661 | 0.014 | 0.001 | 0.027 |
| cPCR, BALF | 0.972 | 0.932 | 0.988 | 0.954 | 0.930 | 0.970 | 710 | 305 | 1652 | 21.178 | 12.438 | 29.918 | 0.030 | 0.004 | 0.056 |
| qPCR, BALF and IS | 0.982 | 0.903 | 0.997 | 0.792 | 0.623 | 0.898 | 210 | 45 | 986 | 4.720 | 1.667 | 7.773 | 0.022 | NE | 0.060 |
| cPCR, BALF and IS | 0.961 | 0.803 | 0.993 | 0.905 | 0.801 | 0.957 | 234 | 49 | 1112 | 10.110 | 2.564 | 17.657 | 0.043 | NE | 0.116 |
| qPCR, IS | 0.980 | 0.944 | 0.993 | 0.815 | 0.721 | 0.883 | 217 | 78 | 601 | 5.303 | 3.024 | 7.583 | 0.024 | 0.000 | 0.049 |
| cPCR, IS | 0.956 | 0.887 | 0.984 | 0.917 | 0.866 | 0.950 | 243 | 90 | 656 | 11.511 | 5.985 | 17.036 | 0.047 | 0.001 | 0.094 |
| qPCR, upper and lower | 0.990 | 0.949 | 0.998 | 0.848 | 0.747 | 0.914 | 536 | 113 | 2541 | 6.529 | 3.033 | 10.025 | 0.012 | NE | 0.032 |
| cPCR, upper and lower | 0.977 | 0.912 | 0.994 | 0.933 | 0.882 | 0.963 | 599 | 156 | 2306 | 14.659 | 6.158 | 23.159 | 0.024 | NE | 0.058 |
| qPCR, URT | 0.892 | 0.710 | 0.965 | 0.905 | 0.809 | 0.955 | 78 | 26 | 238 | 9.340 | 2.997 | 15.682 | 0.120 | NE | 0.245 |
| cPCR, URT | 0.787 | 0.502 | 0.931 | 0.960 | 0.911 | 0.982 | 87 | 27 | 284 | 19.424 | 5.358 | 33.490 | 0.222 | NE | 0.446 |

Sensitivity, specificity, DOR, LR+ and LR- calculated from the coefficients of the binomial regression model. “ll”: lower limit, and “ul”: upper limit of the 95% confidence interval. In the lower limit of LR-, the expression “NE” (not evaluable) appears on some instances. Indeed, the corresponding results obtained by the calculating algorithm had a negative sign, which is impossible as the LRs are ratios between two positive numbers (proportions). This fact is due to the use of an approximate method to calculate the 95% confidence intervals of the LRs, the “delta method”. qPCR; quantitative PCR, cPCR; conventional PCR, BALF; bronchoalveolar lavage fluid, IS; induced sputum, URT; upper respiratory tract, LR; likelihood ratio, sens; sensitivity, spec; specificity, ll; lower limit, ul; upper limit.

**Supplementary 10: Summary Receiver Operating Characteristic (ROC) Plots.**

Bivariate analysis of the sensitivity and specificity of the PCR as a diagnostic tool for PCP. PCP qPCR plots on bronchoalveolar lavage fluid (BALF) samples (A), and induced sputum (IS) samples (B), and upper respiratory tract (URT) specimens (C).

A: Bivariate analysis of the diagnostic accuracy on the 32 studies where the sample was obtained from BALF only.

B: Bivariate analysis of the diagnostic accuracy on the 11 studies where the sample was from IS only.

C: Bivariate analysis of the diagnostic accuracy on the 8 studies where the sample was from URT origin only.
